# A Rechargeable Li-Air Fuel Cell Battery Based on Garnet Solid Electrolytes

**DOI:** 10.1038/srep41217

**Published:** 2017-01-24

**Authors:** Jiyang Sun, Ning Zhao, Yiqiu Li, Xiangxin Guo, Xuefei Feng, Xiaosong Liu, Zhi Liu, Guanglei Cui, Hao Zheng, Lin Gu, Hong Li

**Affiliations:** 1 State Key Laboratory of High Performance Ceramics and Superfine Microstructure, Shanghai Institute of Ceramics, Chinese Academy of Sciences, Shanghai 200050, China; 2 University of Chinese Academy of Sciences, Beijing 100039, China; 3 College of Physics, Qingdao University, Qingdao 266071, China; 4 State Key Laboratory of Functional Materials for Informatics, Shanghai Institute of Microsystem and Information Technology (SIMIT), Chinese Academy of Sciences, Shanghai 200050, China; 5 Qingdao Industrial Energy Storage Research Institute, Qingdao Institute of Bioenergy and Bioprocess Technology, Chinese Academy of Sciences, Qingdao 266101, China; 6 Institute of Physics, Chinese Academy of Sciences, Zhongguancun South 3rd Street No. 8, Beijing 100190, China

## Abstract

Non-aqueous Li-air batteries have been intensively studied in the past few years for their theoretically super-high energy density. However, they cannot operate properly in real air because they contain highly unstable and volatile electrolytes. Here, we report the fabrication of solid-state Li-air batteries using garnet (i.e., Li_6.4_La_3_Zr_1.4_Ta_0.6_O_12_, LLZTO) ceramic disks with high density and ionic conductivity as the electrolytes and composite cathodes consisting of garnet powder, Li salts (LiTFSI) and active carbon. These batteries run in real air based on the formation and decomposition at least partially of Li_2_CO_3_. Batteries with LiTFSI mixed with polyimide (PI:LiTFSI) as a binder show rechargeability at 200 °C with a specific capacity of 2184 mAh g^−1^_carbon_ at 20 μA cm^−2^. Replacement of PI:LiTFSI with LiTFSI dissolved in polypropylene carbonate (PPC:LiTFSI) reduces interfacial resistance, and the resulting batteries show a greatly increased discharge capacity of approximately 20300 mAh g^−1^_carbon_ and cycle 50 times while maintaining a cutoff capacity of 1000 mAh g^−1^_carbon_ at 20 μA cm^−2^ and 80 °C. These results demonstrate that the use of LLZTO ceramic electrolytes enables operation of the Li-air battery in real air at medium temperatures, leading to a novel type of Li-air fuel cell battery for energy storage.

Rechargeable Li-air (Li-O_2_) batteries have attracted intensive research interest in the past few years since they can achieve a much greater energy density than other electrochemical storage devices, which is now in high demand with the rapid development of extended-range electric vehicles and energy storage applications^1^,^2^,^3^,^4^,^5^,^6^,^7^. Li-O_2_ batteries based on non-aqueous electrolytes have been widely investigated because of their rechargeability and potential cycle performance^8^,^9^,^10^,^11^,^12^,^13^,^14^,^15^,^16^,^17^,^18^. However, these batteries face critical challenges for operation in air due to the influences of moisture and carbon dioxide in air, exhausting of the liquid electrolyte in open cells, attacks of peroxide or superoxide on the electrolyte and carbon, sluggish kinetics for oxygen reduction reactions (ORR) and oxygen evolution reactions (OER) at room temperature, as well as dendrite formation of lithium on the anode side. One potential solution is to replace the liquid electrolyte with a solid-state electrolyte^19^,^20^,^21^ that may protect the Li anode and suppress formation of the lithium dendrite, allowing operation of the Li-air battery in real air without destruction of the electrolyte. However, studies on solid-state Li-air batteries (SSLAB) are still in their infancy, mainly due to the lack of available solid electrolytes. So far, several solid-state electrolytes have been used to fabricate SSLAB, such as the NASICON-type lithium-ion conducting ceramics Li-Al-Ge-PO_4_ (LAGP)^22^ and Li-Al-Ti-PO_4_ (LATP)^23^,^24^ and polymer electrolytes such as polyethylene oxide (PEO)^25^. However, inorganic LAGP and LATP ceramic electrolytes become unstable when they come into contact with the lithium metal anode^26^,^27^. As a result, a protective layer must be added to prevent this reaction, which increases the internal resistance of the SSLAB. Polymer electrolytes such as polyethylene oxides (PEO) mixed with LiX (X, anion) are normally unstable at high potentials and are not resistant to the attack of O_2_^−^ species^28^.

A new class of solid-state electrolytes, garnet-type Li_7_La_3_Zr_2_O_12_ (LLZO) ceramics, were first reported by Murugan *et al*.^29^ in 2007. LLZO ceramics have several promising properties, including chemical stability against Li metal, large electrochemical windows (above 5 V), and high Li^+^ conductivity (above 10^−4^ S cm^−1^ at room temperature)^30^. One problem associated with LLZO is the difficulty in sintering high-density ceramics due to the volatility of Li. Recently, through hot-press sintering and optimized processes, we successfully obtained lamellar Li_6.4_La_3_Zr_1.4_Ta_0.6_O_12_ (LLZTO) electrolytes with densities as high as 99.6% and improved the ionic conductivity to 1.6 × 10^−3^ S cm^−1^ at room temperature (as depicted in Figure S1). Based on these LLZTO disk electrolytes (~0.1 cm in thickness), we designed a new type of SSLAB architecture. An air cathode mainly composed of Ketjen black (KB), LLZTO particles and LiTFSI in polyimide (PI:LiTFSI) or polypropylene carbonate (PPC:LiTFSI) was coated on one side of the LLZTO disk. The metallic Li anode was pressed on the other side of the LLZTO disk, which was subsequently sealed airtight. The operating temperature was elevated to improve the interface contact between the Li and LLZTO as well as between the cathode and LLZTO. These two-part SSLABs can be operated in real air with good rechargeability. By replacing PI:LiTFSI with PPC:LiTFSI as the conductive binder, the batteries can be operated at reduced temperatures while displaying improved performance.

## Results

**Figure 1:**
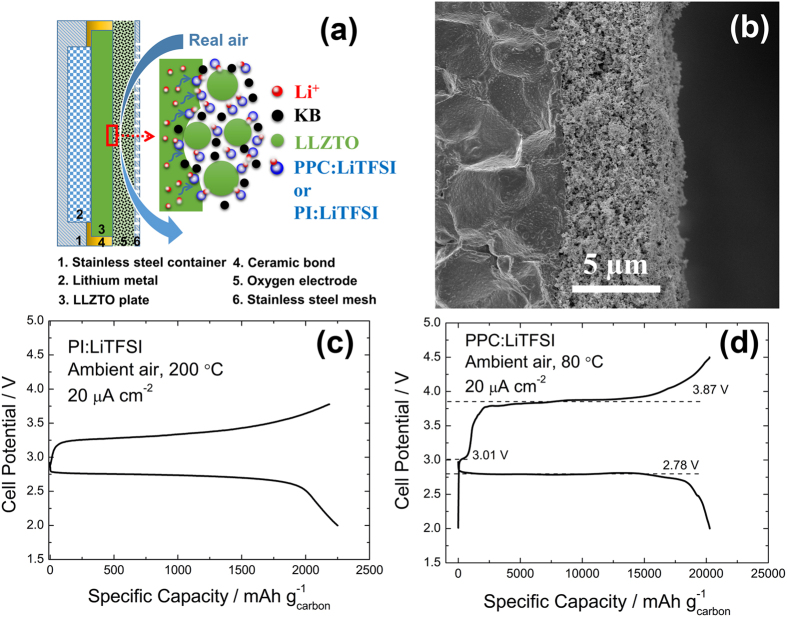
Schematic image and first cycle performance. (**a**) Schematic image and (**b**) cross-section image of a typical SSLAB. (**c**,**d**) Typical first discharge and charge cycles of SSLABs consisting of PI:LiTFSI and PPC:LiTFSI at 200 °C and 80 °C, respectively.

Figure 1a shows the schematic configuration of a typical SSLAB. A 0.1-cm-thick lamellar LLZTO disk is used as the electrolyte. On the anode side, a Li foil is attached on the LLZTO, which is sealed in a stainless steel container with high-temperature resistive sealant. This part can withstand up to 250 °C as indicated by the thermogravimetric-differential scanning calorimetry (TG-DSC) analysis shown in Figure S2. On the cathode side, 0.2-μm LLZTO particles are used as the ion conducting framework. As demonstrated in Figure S3, the plate consisting of LLZTO particles and the PI:LiTFSI composite shows an ionic conductivity of 1.9 × 10^−5^ S cm^−1^ at 25 °C and 3.5 × 10^−5^ at 80 °C, while the PPC:LiTFSI counterpart shows 9.3 × 10^−5^ S cm^−1^ and 1.6 × 10^−4^ S cm^−1^, respectively. The KB used for the cathode is responsible for constructing the electronic network and the occurrence of both ORR and OER^31^. Figure 1b shows a cross-section image of a typical battery. Composition analysis of the air cathode by energy dispersive X-ray analysis (EDX) reveals that La, Zr and C are homogeneously distributed in the whole region (as shown in Figure S4).

Figure 1c shows the discharge and charge behaviours of the batteries with PI:LiTFSI as the binder. These batteries show rechargeability only at 200 °C. Nevertheless, it is worth noting that the coulombic efficiency can be as high as 97.1%, which is likely due to the influence of elevated temperature on the Li anode and the interfacial resistance. To lower the operation temperature of the batteries, PI:LiTFSI is replaced with PPC:LiTFSI, which can reduce the interfacial resistance as discussed above. The resulting batteries can be cycled at 80 °C with a current density of 20 μA cm^−2^, as shown in Fig. 1d. After a large discharge plateau at approximately 2.78 V, the curve drops suddenly at a specific potential. The specific discharge capacity reaches approximately 20300 mAh g^−1^_carbon_, which is approximately 6430 mAh g^−1^_cathode_ when being calculated according to the overall cathode mass. Upon charging, a plateau at 3.01 V with capacity of approximately 650 mAh g^−1^_carbon_ is initially observed. Then, a large plateau at 3.87 V with a capacity of approximately 15000 mAh g^−1^_carbon_ appears. To clarify the origin of the charge reaction at 3.87 V, the composites with PPC:LiTFSI and KB as the cathodes were charged in air at 80 °C (curve displayed in Figure S5). These composites display decomposition only when the charge potential is greater than 4.45 V. This indicates that the capacity during charging at 3.87 V is solely related to decomposition of the discharge products rather than decomposition of the composite cathode.

**Figure 2:**
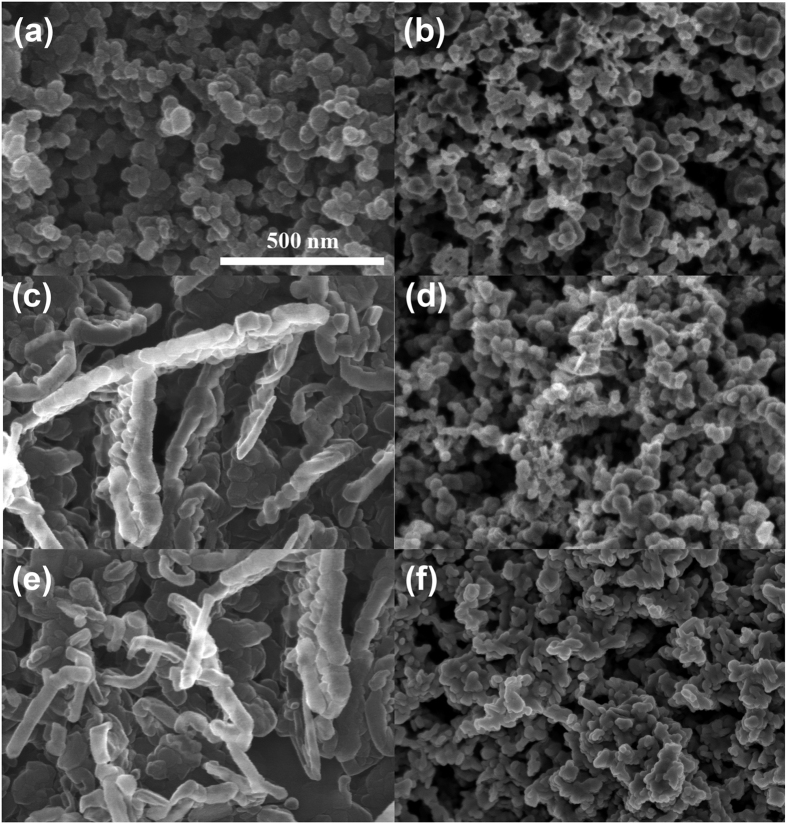
Morphology of the air cathode. (**a**) Pristine air cathode, (**b**) first discharge to a capacity of ∼10000 mAh g^−1^_carbon_, (**c**) first discharge to a capacity of ∼20000 mAh g^−1^_carbon_ (~2.0 V), (**d**) first charge to a capacity of 20000 mAh g^−1^ (~4.5 V), (**e**) fifth discharge to 2.0 V, and (**f**) fifth charge to 4.5 V at 20 μA cm^−2^. The white scale bar represents 500 nm for all images.

The morphology and composition of the reaction products were characterized by both *ex-situ* and *in-situ* techniques. Figure 2 shows SEM images of changes in the air cathode during cycling. Compared to the pristine cathode (Fig. 2a), the average size of granular particles increases at the half discharge state (Fig. 2b). After full discharge (Fig. 2c), rod-like particles can be observed, indicating that the discharge products grow upward and merge with each other to form agglomerated particles^32^. Upon charging, the large particles identified in Fig. 2c clearly shrink, as shown in Fig. 2d. After the fifth discharge (Fig. 2e), rod-like particles similar to those formed at the initial discharge (Fig. 2c) and during the shrinkage (Fig. 2f) can also be observed, indicating repetitive formation and decomposition of the discharge product.

**Figure 3:**
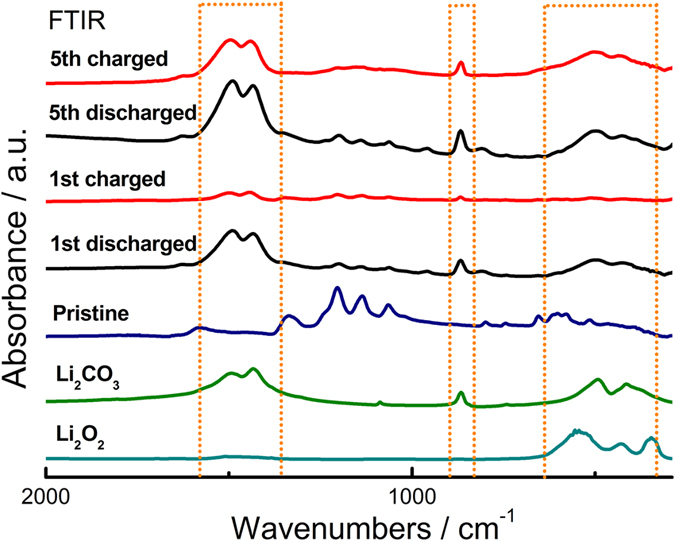
FTIR measurement. FTIR spectra of air cathodes after the 1 st and 5th discharge (black lines) and 1st and 5th charge (red lines) at 80 °C. Regions are highlighted by the dotted lines corresponding to the peaks of Li_2_CO_3_. The spectra of pristine air cathodes, pure Li_2_CO_3_ (olive line) and Li_2_O_2_ (dark cyan line) powders are provided for comparison.

Fourier transform infrared spectroscopy (FTIR) was also performed on the air cathodes after discharge and charge processes at 80 °C. Considering that PPC has complex FTIR peaks between 2000 cm^−1^ and 1250 cm^−1^ 33, where the characteristic peaks of Li_2_O_2_ and Li_2_CO_3_ lie, we used the batteries with PI:LiTFSI in the FTIR measurement. As shown in Fig. 3, the peak intensity corresponding to the Li_2_CO_3_ reference greatly increases after discharge, which indicates that the identified discharge product is Li_2_CO_3_. The same phenomenon was observed by transmission electron microscopy (TEM) of the air cathodes, in which carbon nanotubes (CNT) were used as the active material instead of KB to clearly characterize the product composition upon cell operation. As shown in Figure S6, the Li_2_CO_3_ grows around the CNT during discharging and decomposes upon charging.

**Figure 4:**
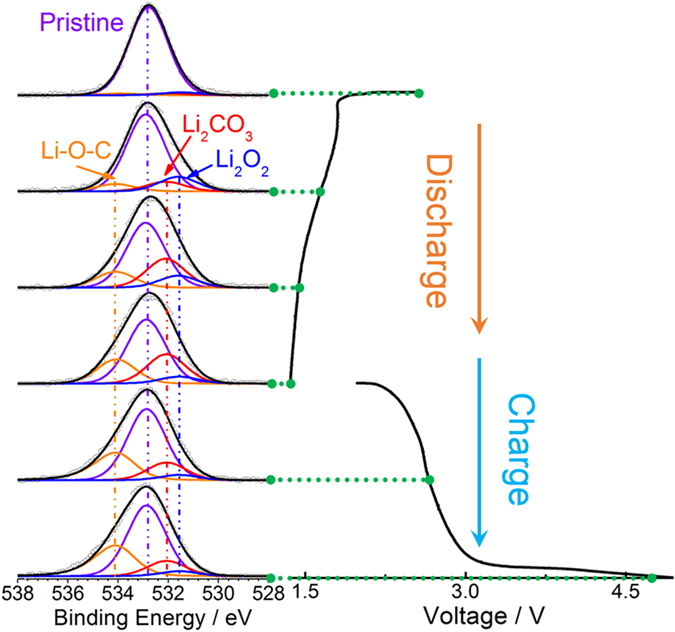
*In-situ* XPS analysis. *In-situ* XPS of O 1s collected under air atmosphere of 10^−4^ atm for the carbon cathodes during the first discharge/charge cycle of the SSLAB cell.

To obtain more information on the change in chemical composition on the cathode surfaces, *in-situ* X-ray photoelectron spectroscopy (XPS) of the discharge and charge process was performed under ambient pressure of 0.1 mbar. The cathodes used in the *in-situ* XPS measurement were carbon materials that were deposited on the LLZTO disk by sputtering (details can be found in Methods section). As shown in Fig. 4, Li_2_O_2_ peak intensity first increases and then decreases upon discharge. Meanwhile, the Li_2_CO_3_ and Li-C-O peak intensities steadily increase with increasing depth of discharge. These results indicate that the Li_2_O_2_ is formed at the initial stage of discharge, and reacts with trace H_2_O and CO_2_ in ambient air to form a large quantity of Li_2_CO_3_ on the cathode surfaces. The appearance of the Li-O-C peak can be attributed to the decomposition of the carbon cathode (i.e., KB) when the cell is charged, according to ref. 34. Note that *in-situ* measurement allows the observation of transient products (i.e., Li_2_O_2_)^35^,^36^. The *ex-situ* measurement can only provide information on the stabilized products in air, such as Li_2_CO_3_. While charging, the peak intensity of Li_2_CO_3_ greatly decreases, indicating that the Li_2_CO_3_ is most probably decomposed upon charging, at least partially. Rechargeable Li-CO_2_ batteries based on the formation and decomposition of Li_2_CO_3_ have already been demonstrated in previous reports^37^,^38^, which also indicated that the decomposition of Li_2_CO_3_ upon charge was dependent on the type of carbon materials in the cathodes. The KB carbons used here are most likely not the optimum components, causing partial decomposition of Li_2_CO_3_ upon charge and thus accumulation of passivated Li_2_CO_3_ products after multiple cycles.

Based on the existing data and previous reports, we propose the following reaction route in the present Li-air battery. During discharging, O_2_ is electrochemically reduced to form Li_2_O_2_ (equation 1) in the cathode initially. Li_2_O_2_ then reacts with CO_2_ in air to form Li_2_CO_3_ via a chemical reaction (equation 2). During charging, Li_2_CO_3_ is electrochemically decomposed at least partially, according to the release of CO_2_ during charge (as shown in Figure S8).


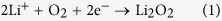



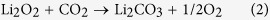


In fact, preliminary DEMS (differential electrochemical mass spectrometry) results indicate that the reaction mechanism upon charging is very complicated. Further work with isotope labelling is necessary and ongoing in our laboratory.

**Figure 5:**
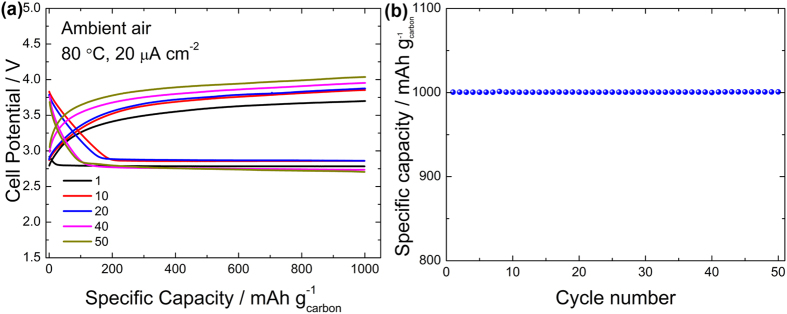
Cycle performance of SSLAB. (**a**) The PPC:LiTFSI cell operated at 80 °C in real air with a cutoff discharge capacity of 1000 mAh g^−1^_carbon_ at 20 μA cm^−2^ and (**b**) the specific capacity as a function of cycle number.

According to the above analysis, the discharge products grow on the cathode surfaces during discharge and partially decompose during charging. This situation is similar to Li-O_2_ batteries based on Li_2_O_2_ formation and decomposition^39^. The insulating deposits have a passivating influence on active sites for ORR^40^,^41^. In the case of full discharge, a number of thick discharge products form and coat the carbon surfaces, which allows only a few cycles, as shown in Figure S7. To increase the cycle number, limiting the depth of discharge is necessary. As shown in Fig. 5a, the batteries can be run for more than 50 cycles at 20 μA cm^−2^ when the discharge capacity is cut off at 1000 mAh g^−1^_carbon_, which is approximately 316 mAh g^−1^_cathode_ if calculated according to the overall cathode mass.

## Discussion

Here we demonstrate the fabrication of rechargeable SSLABs that can be operated in real air at elevated temperatures. The newly synthesized LLZTO ceramic electrolytes play essential roles in these batteries. They are dense (99.6% in relative density), highly conducting (1.6 × 10^−3^ S cm^−1^ at room temperature)^42^, and are able to prevent gas species from diffusing through and reacting with the Li anodes. LLZTO ceramic electrolytes are also chemically and electrochemically stable against Li^29^,^43^,^44^, which allows direct contact with Li without introducing an additional resistive intermediate layer. Previous reports found that LLZTO might react with H_2_O in air, causing degradation of ionic conductivity^45^,^46^, but the moisture content in air can be greatly reduced when the surrounding temperature is elevated to above 80 °C^47^. This means that the problem of LLZTO degradation can be greatly relieved at elevated temperatures.

Other important issues include construction of the air cathode and interfacial concerns. Air cathodes cannot be wetted in the solid-state electrolytes as they do in the liquid electrolytes. As a result, it is necessary to artificially build the electronic and ionic network. Because the discharge products are solids deposited on the cathode surfaces, sufficient space to accommodate these solids is also required for large capacities, which is why porous composite cathodes are needed. The LLZTO particles provide a framework that is ionically conducting and able to create space for air diffusion, while PPC:LiTFSI enhances ion conduction at the LLZTO interfaces.

The use of elevated temperatures is especially advantageous in terms of improving ion conduction at the Li/LLZTO and electrode/LLZTO interfaces. However, too high operating temperature may cause the decomposition of carbon-based cathodes. As a result, selecting suitable carbon materials, and optimizing cathode configuration to reduce the interfacial resistance must be considered to improve battery performance. Moreover, searching for suitable materials with effective catalysts that can promote the decomposition of Li_2_CO_3_ upon recharge is also crucial for the development of SSLABs.

## Conclusions

A novel type of solid-state Li-air battery based on LLZTO ceramic electrolytes is demonstrated here. After optimizing the interfacial resistance, the batteries show a specific capacity of approximately 20300 mAh g^−1^_carbon_ at 20 μA cm^−2^ (or 6430 mAh g^−1^_cathode_ if referring to the mass of cathode) in real air at 80 °C. With a cutoff discharge capacity of 1000 mAh g^−1^_carbon_ at 20 μA cm^−2^, the batteries can be operated for 50 cycles while maintaining their initial capacity. Analysis of the cell chemistry reveals that the batteries operate through formation and decomposition of Li_2_CO_3_ at least partially. Cathode design related to the interfacial issue and materials research to promote decomposition of the discharge product are two key problems worth investigating in the near future. The performance achieved in the SSLABs presented here demonstrates the possibility of operating Li-air batteries in real air, which opens new avenues for the use of Li-air fuel cell battery for energy storage.

## Methods

### Material preparation and cell assembly

LLZTO powders in the cubic phase were prepared via a conventional solid-state reaction. More details can be found in our previous paper^42^. The bulk ceramics were fabricated in lamellar disks with a diameter of 1.2 cm and a thickness of 0.1 cm by the hot-press sintering technique.

Composite cathodes consisted of Ketjen black (KB), polyimide (PI) or polypropylene carbonate (PPC), Li(CF_3_SO_2_)_2_N (LiTFSI) (99.95%, Sigma-Aldrich, Shanghai, China), and LLZTO powders (D_50_~200 nm). The PI suspension was prepared by dispersing PI powder in N-methyl-2-pyrrolidone (NMP) and was stirred before use; the PPC solution was prepared by dissolving PPC in NMP. LiTFSI was dissolved in NMP before mixing with KB and LLZTO powders in an agate mortar and grinding for 1 h. The obtained slurries were coated on one side of the LLZTO ceramic membrane by blades, then pre-dried in an oven at 80 °C for 2 h to remove NMP and baked at 80 °C in vacuum overnight. The typical loading mass of each cathode was approximately 1 mg cm^−2^. The weight ratio between KB, LLZTO, LiTFSI/and PI or PPC was fixed at 6.3:6.3:6.3:1. Li anodes were attached on the other side under high pressure in an Ar-filled glove box with oxygen and moisture levels below 0.1 ppm. The Li-side was sealed in a stainless steel container with high-temperature resistive sealant. Stainless steel (SS) foils were selected as current collectors. Both the specific capacity and the current density were normalized to the mass of KB.

### Electrochemical measurement

Galvanostatic charge and discharge behaviours of the batteries were investigated using an Arbin battery cycler at 80 °C or 200 °C that was calibrated by a thermocouple attached to the measured cell. Before testing, the cells were first rested in a thermostatic oven for 6 h to reach equilibrium. Electrochemical impedance spectroscopy (EIS) was performed using an Autolab instrument.

### Characterization

Crystalline structures of the LLZTO powders and ceramics were examined by X-ray diffraction (XRD, Bruker D2 Phaser) using Cu Ka radiation. Ionic conductivities of LLZTO ceramics were measured using an impedance analyzer (Novocontrol Beta High Performance Impedance Analyzer) at a temperature range of 20 to 100 °C with frequency ranging from 0.1 to 20 MHz with an amplitude of 10 mV. Surface and cross-section morphologies of the LLZTO ceramics and the cathodes were analyzed by scanning electron microscopy (SEM, FEI Magellan 400, Hitachi S-4800). High-resolution transmission electron microscopy (HR-TEM, JEOL JSM-6700F) and selected area electron diffraction (SAED) measurements were performed to investigate the solid products.

### *In-situ* XPS

XPS data were collected from the top cathode surface of the SSLAB under air atmosphere during cell operation at *SIMIT* 35. The cell was placed onto an insulating sample holder. The air cathodes were carbon materials, which were coated on the LLZTO disks by sputtering. The bottom and surface sides of the cells were in contact with two silver wires that served as current collectors. Two copper cables were used to connect the negative and positive electrodes to an external Bio-Logic SP-300 potentiostat. No other precleaning was performed. XPS data were collected under an atmosphere of 10^−4^ atm at an operating temperature of 80 °C with a current density of 20 μA cm^−2^. The cell was discharged and charged for 6 h each. XPS spectra were collected at a photon energy of 1486.6 eV. The binding energy of all the spectra was calibrated to the Pt 4 f peak of sputtered Pt foil. Shirley background subtraction was applied to the photoemission lines, which were fitted using a combined Gaussian-Lorentzian line shape (CasaXPS).

## Additional Information

**How to cite this article**: Sun, J. *et al*. A Rechargeable Li-Air Fuel Cell Battery Based on Garnet Solid Electrolytes. *Sci. Rep.* **7**, 41217; doi: 10.1038/srep41217 (2017).

**Publisher's note:** Springer Nature remains neutral with regard to jurisdictional claims in published maps and institutional affiliations.

## Supplementary Material

Supplementary Information
