# Supplementary material for: A Rechargeable Li-Air Fuel Cell Battery Based on Garnet Solid Electrolytes
Source: Sci Rep. 2017 Jan 24;7:41217. doi: 10.1038/srep41217 (PMC5259739; doi:10.1038/srep41217)
Supplement: Supplementary Information [file srep41217-s1.pdf]

Supporting Information

A Rechargeable Li-Air Fuel Cell Battery Based on Garnet Solid Electrolytes

Jiyang Sun,[a,b,1] Ning Zhao,[c,1] Yiqiu Li,[a] Xiangxin Guo,[a]† Xuefei Feng,[d] Xiaosong Liu,[d] Zhi Liu,[d] Guanglei Cui,[e] Hao Zheng,[f] Lin Gu,[f] Hong Li[f]

[a] State Key Laboratory of High Performance Ceramics and Superfine Microstructure, Shanghai Institute of Ceramics, Chinese Academy of Sciences, Shanghai 200050, China.

[b] University of Chinese Academy of Sciences, Beijing 100039, China.

[c] College of Physics, Qingdao University, Qingdao 266071, China

[d] State Key Laboratory of Functional Materials for Informatics, Shanghai Institute of Microsystem and Information Technology (SIMIT), Chinese Academy of Sciences, Shanghai 200050, China.

[e] Qingdao Industrial Energy Storage Research Institute, Qingdao Institute of Bioenergy and Bioprocess Technology, Chinese Academy of Sciences, Qingdao 266101, China.

[f] Institute of Physics, Chinese Academy of Sciences, Zhongguancun South 3rd Street No. 8, Beijing 100190, China.

† Corresponding author: Tel: +0086 21 52411032; Fax: +0086 21 52411802. E-mail address: xxguo@mail.sic.ac.cn.

[1] The authors contributed equally to this work.

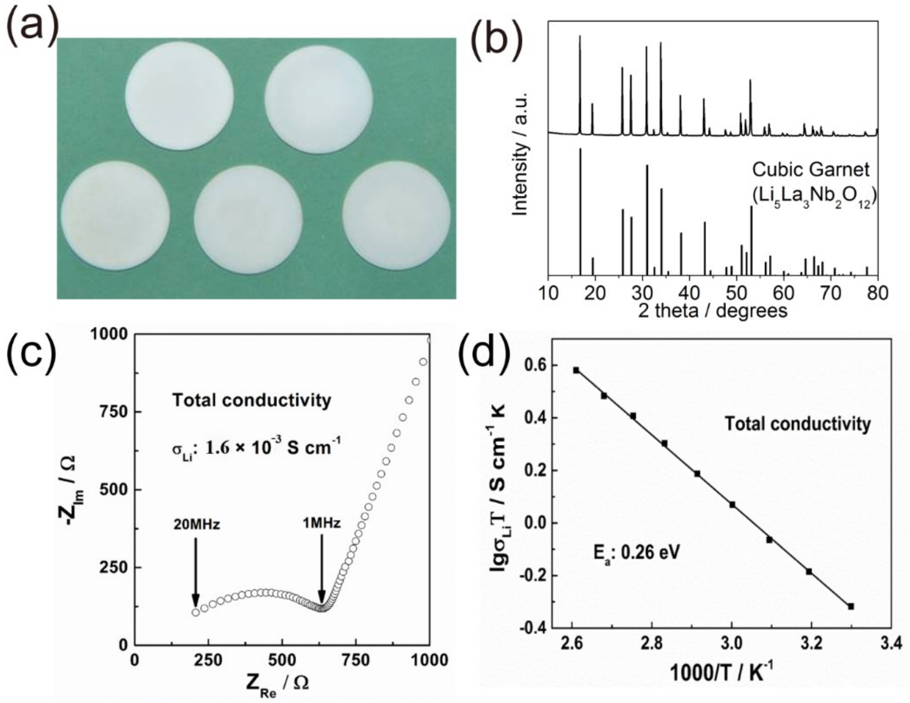

Figure S1. (a) Photos for the lamellar LLZTO ceramics with thickness of approximately 0.1 cm and diameter of 1.2 cm; (b) The XRD patterns of the LLZTO ceramics and the cubic garnet $Li_5La_3Nb_2O_{12}$ (JCPDS 01-080-0457) as a reference; (c) Impedance spectroscopy measured at 25°C for the LLZTO ceramics with Au electrodes; (d) The Arrhenius plot for the above ceramics. The relative density of ceramic plate is as high as 99.6%, and the corresponding ionic conductivity is 1.6×10^{-3} S cm^{-1}at 25 °C.

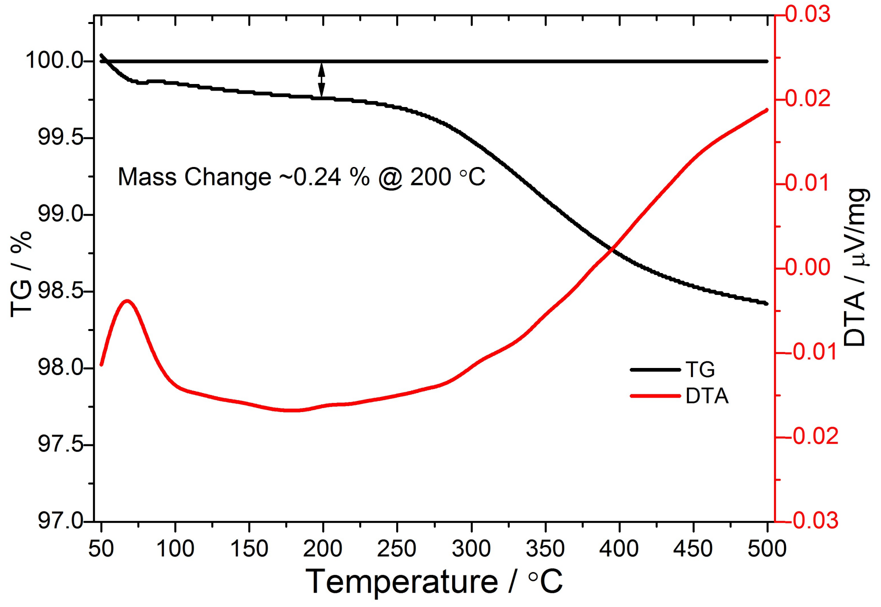

Figure S2. Thermogravimetric-differential scanning calorimetry (TG-DSC) analysis of high-temperature resistive sealant, indicating mass change less than 0.3% up to 250 °C.

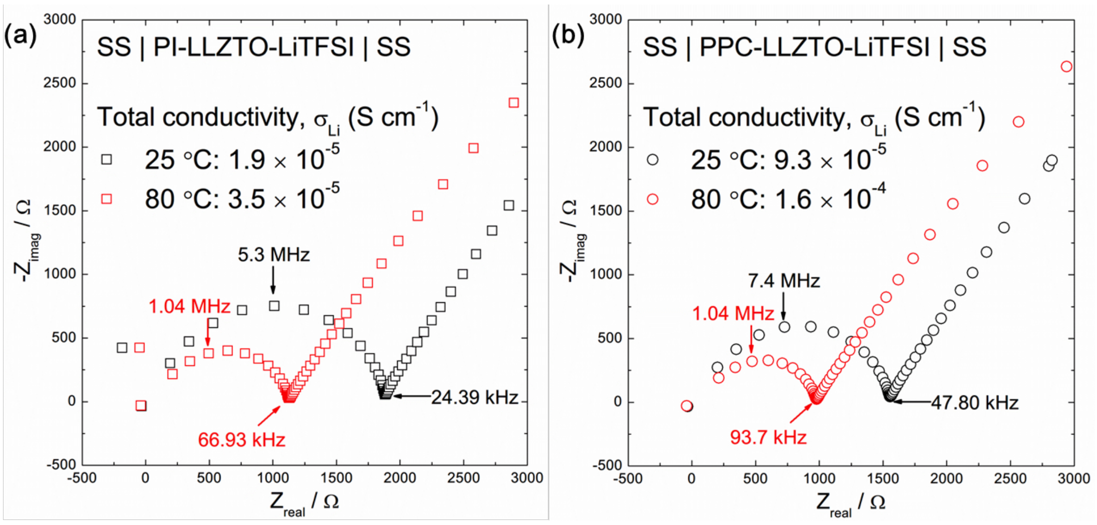

Figure S3. Impedance spectroscopy of the pellet consisting of LLZTO particles and (a) PI:LiTFSI or (b) PPC:LiTFSI composite. The electrodes used in the measurement are stainless steel (SS). It is calculated that the corresponding ionic conductivity is approximately 1.9×10^{-5} S cm^{-1} at 25 °C and 3.5×10^{-5} S cm^{-1} at 80 °C for PI:LiTFSI, while 9.3×10^{-5} S cm^{-1} and 1.6×10^{-4} S cm^{-1} for PPC:LiTFSI.

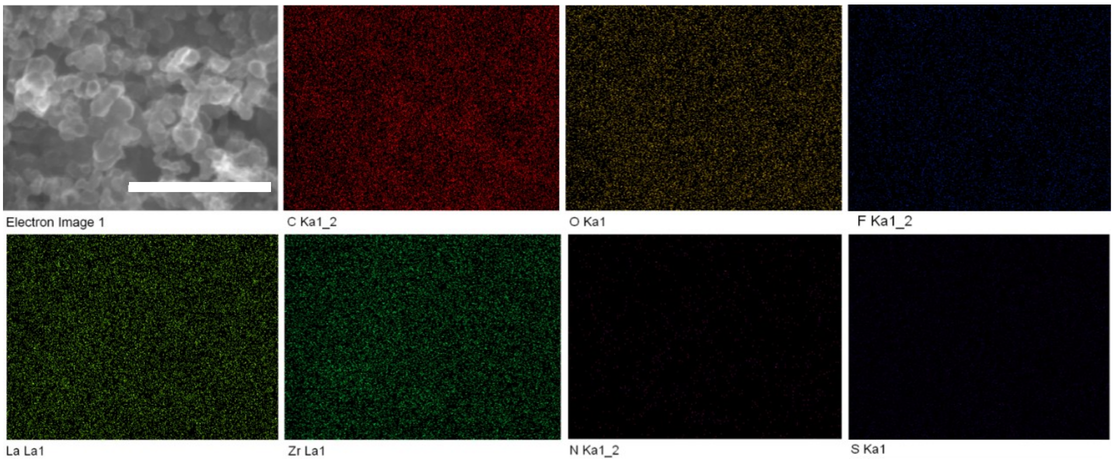

Figure S4. Composition distribution of the air cathode by energy dispersive x-ray analysis, indicating that the elements of La, Zr, O, C, F, N, S homogeneously distribute in the whole range of composite air cathode.

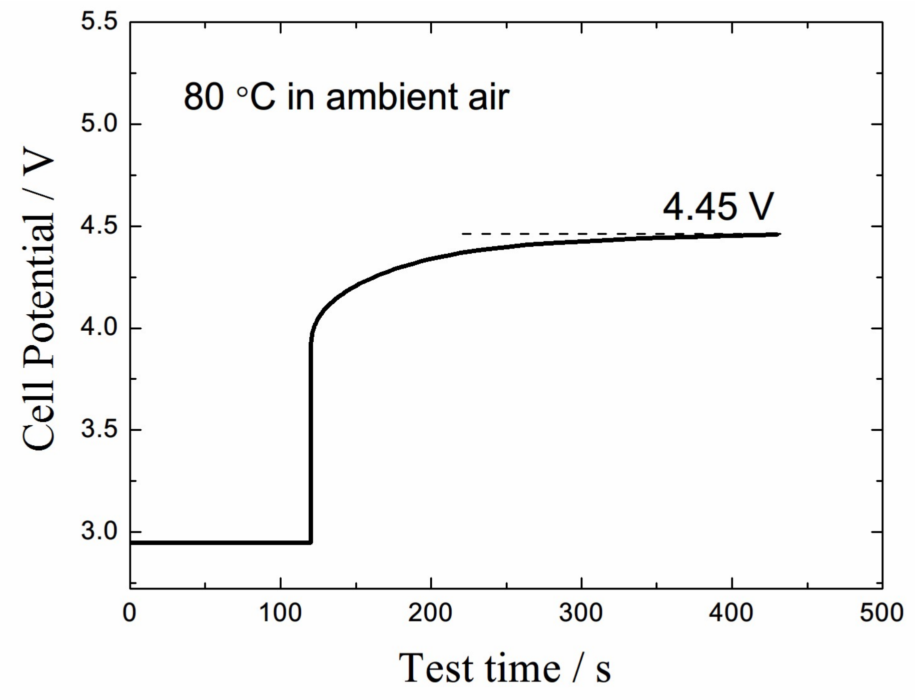

Figure S5. Charge curve of solid-state Li-air batteries with KB-LLZTO-PPC:LiTFSI cathode without pre-discharge.

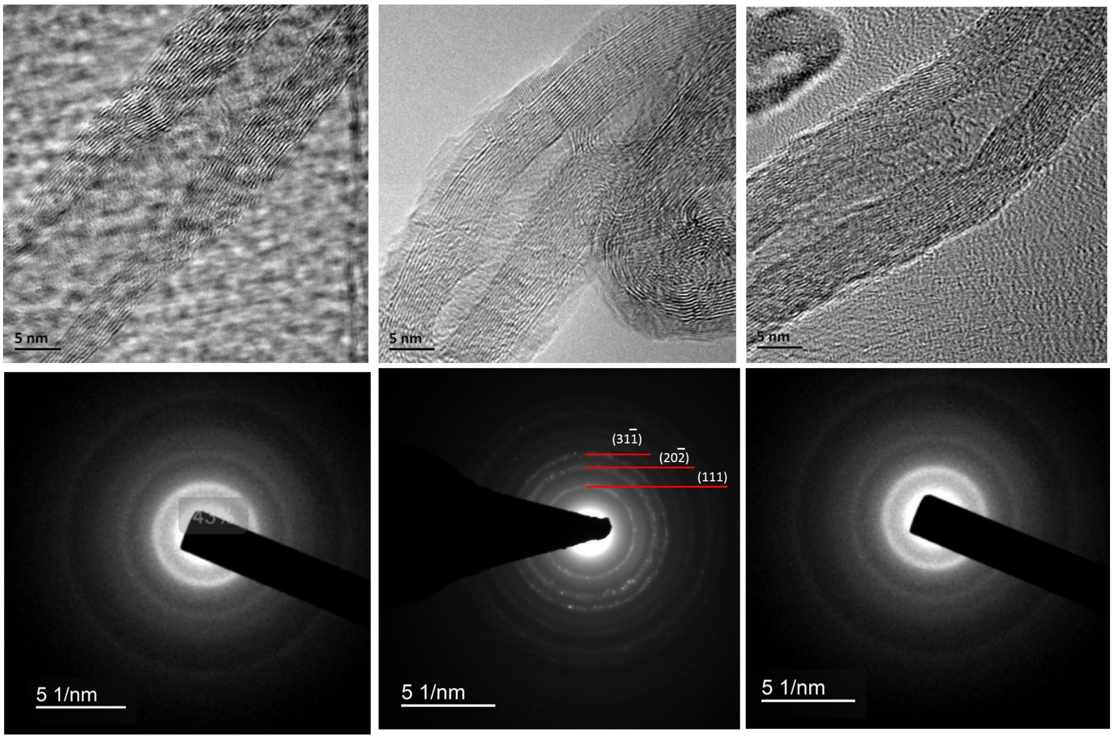

Figure S6. (a) High resolution TEM images of discharged CNT air cathodes at 10 mA g^{-1}. (b) shows the CNT morphology after discharge and (c) shows that after charge. The corresponding selected area electron diffraction (SAED) patterns of the pristine, discharged and charged cathodes are given in (d), (e), (f), respectively. In (e), the measured d-spacings can be well indexed to the diffractions of Li_2CO_3 (JCPDS card no. 22–1141), and other weak rings can be indexed to diffraction of CNT.

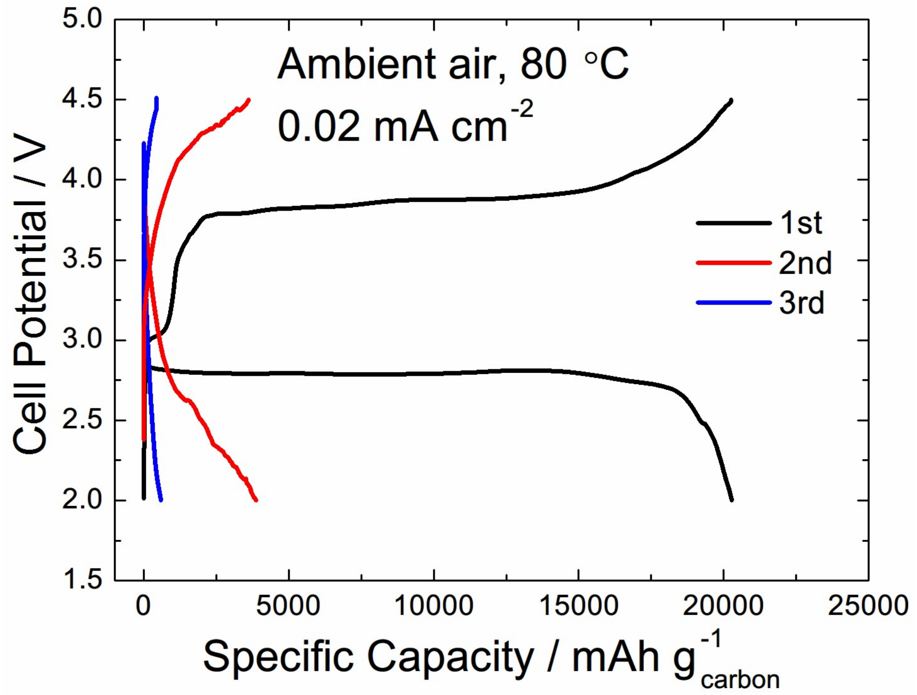

Figure S7. Cycle performance of solid-state Li-air battery with full discharge and charge under ambient air at 0.02 mA cm^{-2} and 80 °C.

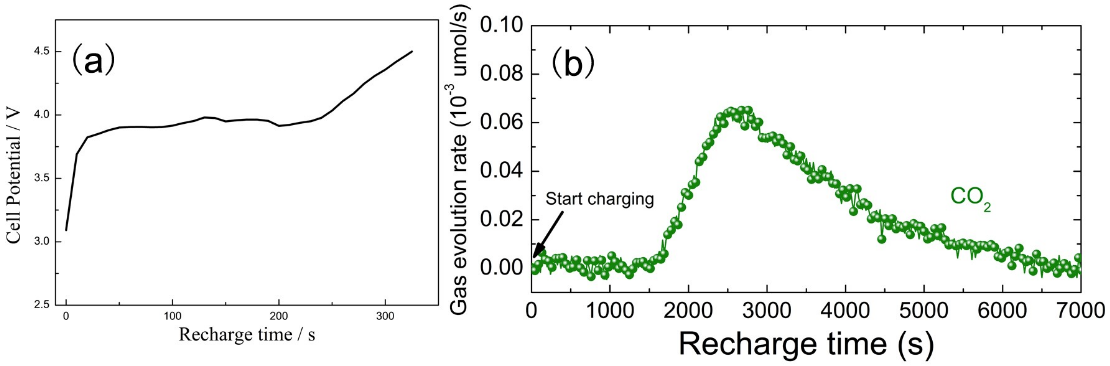

Figure S8. *In-situ* DEMS (differential electrochemical mass spectrometry) measurement of solid-state Li-air battery with KB-LLZTO-PPC:LiTFSI composite cathode during charging. The cell was first discharged in ambient air at 80 °C and transferred into a customized Swagelok cell for DEMS measurementt in an Ar-filled glove box. Ultrapure Ar was used as carrier gas. The DEMS measurement was carried out with the help of Prof. Zhangquan Peng. (Changchun Institute of Applied Chemistry, Chinese Academy of Science)
